# Role of microbiota in the outcome of immune checkpoint inhibition therapy of cancer

**DOI:** 10.37349/etat.2025.1002348

**Published:** 2025-11-18

**Authors:** Ger T. Rijkers, Yonah Langcauon, Pippe van Leersum, Lara Popović, Frans J. van Overveld

**Affiliations:** IRCCS Istituto Romagnolo per lo Studio dei Tumori (IRST) “Dino Amadori”, Italy; Department of Health, Cognition, and Behavior, University College Roosevelt, Middelburg 4330 CB, The Netherlands

**Keywords:** immune checkpoint inhibition therapy, gut microbiota, regulatory T cells, tumor residing bacteria

## Abstract

The realization that the composition and functionality of gut microbiota have an impact on the outcome of immune checkpoint inhibition (ICI) therapy of cancer has initiated research into the potential of microbiota management as adjunctive therapy. Fecal microbiota transplantation can improve the outcome of ICI, but for optimal donor selection, safety, and large-scale implementation, there remain bottlenecks. Alternative strategies, such as the use of selected bacterial species, require fundamental knowledge of the underlying mechanisms governing the interaction between (intestinal) microbiota and the immune system. Gut microbiota also appears to be able to colonize the tumor microenvironment. Some bacterial species directly or indirectly promote tumor growth. Other defined species have tumoricidal properties. These findings and insights are now being used to further optimize the functionality of the immune system and shape the tumor microenvironment in order to improve the outcome of ICI.

## Introduction

Tumor cells can evade the immune system in many different ways. One of the most important is the expression of checkpoint inhibition molecules such as PD-ligand 1 (PD-L1). The realization of the crucial role of checkpoint inhibition, and thus the potential for new ways of therapy of cancer, has sparked research into in vivo application of monoclonal antibodies against PD-L1 and other checkpoint inhibitors, either on the tumor cells or on cytotoxic T lymphocytes [1–3]. This novel form of cancer immunotherapy, immune checkpoint inhibition (ICI) therapy, turned out to be a breakthrough for which James Allison and Tasuku Honjo, because of their fundamental discoveries of checkpoint molecules, received the Nobel prize in 2018 [4, 5]. For a number of cancers, even when metastasized, ICI therapy has been effective, but unfortunately, not in all patients. The actual eradication of the tumor depends on the functionality of the cellular immune system of the patient, which is likely the reason for the qualified success.

The cover story of *Science* magazine of January 5, 2018, was on Gut Microbes and Cancer, with the subheading “Intestinal microbiota influence cancer patient responses to immunotherapy” [6]. The evolving understanding of the mechanism underlying this effect is that gut microbiota, either residing in the intestine or having translocated to the tumor microenvironment, modulate the immune system of the patient, enabling a successful anti-tumor immune response. A full understanding of the cellular and molecular interactions could ultimately lead to precise microbiota management and thus further improvement of ICI therapy of cancer.

## Immune checkpoint inhibition therapy and microbiota

The first indication that (gut) microbiota can have an impact on the outcome of ICI therapy came from the seminal study by Routy et al. [7] in 2018. These authors found that the outcome of ICI therapy in patients with non-small cell lung carcinoma (NSCLC), who were treated with antibiotics in the period directly before programmed death 1 (PD-1)/PD-L1 targeted ICI therapy, was significantly poorer than in patients not treated with antibiotics [7]. Moreover, analysis of the gut microbiota composition of the patients showed a significant association between the clinical responses to ICIs and the relative abundance of a specific bacterium, *Akkermansia muciniphila* (*A. muciniphila*). In germ-free mice, inoculated with a fibrosarcoma cell line, tumor growth could be inhibited with PD-1 blockade only when the mice were given a fecal microbiota transplantation (FMT) with fecal material from patients who previously had responded (R) to ICI therapy, but not with an FMT from non-responder (NR) patients. Remarkably, the addition of *A. muciniphila* to FMT from NR patients also restored the ability of the mice to respond to the ICI treatment. Unfortunately, later studies, discussed below, showed that *A. muciniphila* is not the “magic bacterial bullet” that renders ICI therapy successful in every patient [7, 8].

Within 5 years after the above-described human studies and animal experiments, Routy et al. [9] published a new study expanding on their previous findings. This study used FMT plus anti-PD-1 ICI in a phase 1 trial in patients with advanced melanoma. Of the 20 patients included in the study, none were previously treated with anti-PD-1, and the fecal material for the FMT was taken from three healthy male donors. After a follow-up of over 20 months, the median progression-free survival had not been reached, with 16 patients surviving. These data showed that FMT, using fecal matter from healthy donors, is safe for patients in the first-line setting.

Many more clinical studies on the effect of FMT on the outcome of ICI therapy have subsequently been initiated. Baruch et al. [10] found that 3 out of 10 patients, previously unresponsive to anti-PD-1 ICI, had partial and complete responses after having had FMT. Notably, two different fecal donors, who had previously been treated with anti-PD-1 monotherapy and had achieved a complete response of at least 1 year, were used of which only one donor was responsible for the 3 patients gaining a response. This effect indicates the importance of the composition of the fecal microbiota, but the reason for the dissonance between donor 1 and donor 2 is unclear. Donor group 1 showed a higher relative abundance of taxa like *Bifidobacterium adolescentis*, whereas donor group 2 had a high relative abundance of taxa like *Ruminococcus bromii*. Moreover, a higher relative abundance of Enterococcaceae, *Enterococcus*, and *Streptococcus australis,* and a lower relative abundance of *Veillonella atypica* in both donor groups [10]. However, the analysis of these specific taxa across the entire patient cohort revealed that some NRs and pretreatment samples displayed similar microbial dynamics. As a result, no definitive association between these bacterial classes and clinical response to therapy could be established from this study. The heterogeneity of FMT efficacy is therefore not fully understood yet, but must include both donor-specific effects, such as microbiota composition, as well as host factors.

Moreover, another phase 1 trial showed that 6 out of 15 patients, with an immunological ability to respond to the treatment and an unfavorable microbiota composition, responded to responder-derived FMT with anti-PD-1 treatment [11]. Some discussion worthy findings were that in this study the microbiota of complete responding donors displayed greater alpha diversity compared to partial responding donors. Additionally, gut bacterial commensals previously associated with enhanced responses to anti-PD-1 therapy, such as *Faecalibacterium prausnitzii* and *A. muciniphila*, exhibited a negative correlation with CXCL8 (IL-8). In contrast, members of the *Bacteroides genus*, which have been linked to reduced responses to anti-PD-1 treatment, showed a positive correlation with CXCL8 (IL-8).

### Mechanisms underlying the improvement of the outcome of ICI therapy by gut microbiota

While the studies highlighted above have shown an association between gut microbiota composition and the outcome of ICI therapy, they do not suggest potential mechanisms. The remodeling of gut microbiota via FMT improves the outcome of ICI, but not in all patients. FMT, even when using fecal material from super donors, does not guarantee success in every patient. To improve the outcome of ICI therapy, a better understanding of the interactions between microbiota and the immune system is needed. Based on previous gut microbiota and probiotic studies, it is known that gut microbiota can modulate the functionality of the immune system.

#### Immune modulation by gut microbiota

Fermentation of dietary fibers and indigestible carbohydrates by gut microbiota leads to the production of short-chain fatty acids (SCFAs). The main SCFAs are butyrate, acetate, and propionate. An important target cell is the regulatory T cell (Treg), a cell characterized by expression of CD25 and the transcription factor forkhead box P3 (FOXP3). Tregs maintain and restore the balance between Th1, Th2, Th17, and Tfh cells [12]. It has been extensively demonstrated that the induction of Tregs is mediated by butyrate, produced by gut microbiota. Butyrate enhances histone H3 acetylation in the promoter region of the (FOXP3) locus [13]. FMT leads to an increase in CD25^+^FOXP3^+^ Treg and concomitantly reduces the number and activity of Th17 cells and related cytokines IL-6 and IL-17 [14].

The induction of Tregs and repression of Th17 cells is the preferred type of immunomodulation in the case of inflammatory diseases as well as allergic diseases [15]. In the case of tumor immunology, activation of Tregs would be undesired [16]. Tumor tissue consists of the tumor cells but also an array of non-tumor cells, including invading cells of the innate and acquired immune system. Collectively, this is defined as the tumor microenvironment (TME). Within the TME, activation of Tregs can best be avoided. The TME contains different subsets of Tregs, including thymic-derived Tregs (tTregs), peripheral induced Tregs (pTregs), tissue-resident Tregs (tr-Tregs), and follicular Tregs (Tfr), but they all have immunosuppressive effects, therefore promoting tumor immune evasion. Indeed, it was found that patients with multiple myeloma or metastatic prostate carcinoma treated with anti-CTLA-4 responded significantly poorer to therapy than patients with high serum butyrate levels and higher numbers of blood Tregs [17].

**Figure 1:**
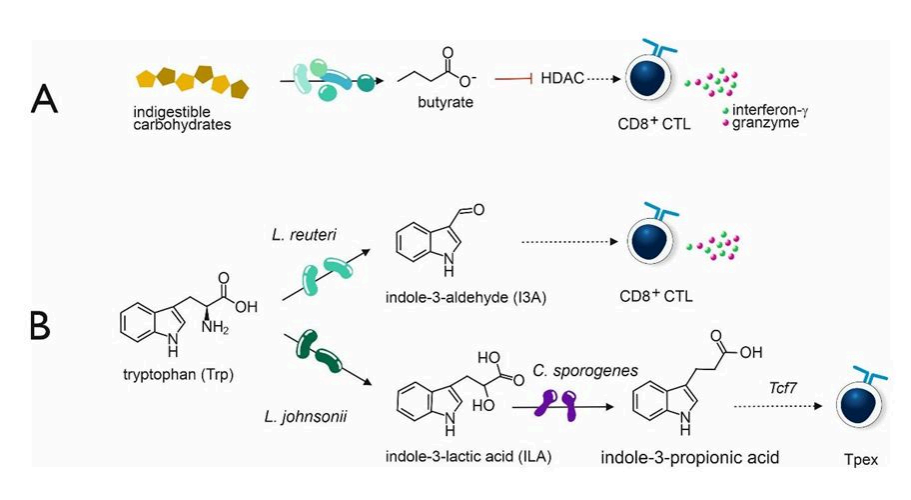
**Bacterial metabolites involved in the anti-tumor response.** (**A**) Butyrate stimulates the tumor-killing capacity of CD8^+^ cytotoxic T cells (CTL). (**B**) The bacterial metabolites are generated from tryptophan (Trp) metabolism. *Lactobacillus reuteri* (*L. reuteri*) converts Trp into indole-3-aldehyde (I3A), which has a stimulatory effect on CTLs. *Lactobacillus* johnsonii (*L. johnsonii*), along with *Clostridium sporogenes* (*C. sporogenes*), converts Trp into indole-3-propionic acid (IPA). This molecule activates the transcription factor gene *Tcf7*, which contributes to the generation of precursor exhausted T cells (Tpex), precursors of effector CTLs. HDAC: histone deacetylase. Adapted from [23]. ©2025 The authors. Licensed under a CC BY (https://creativecommons.org/licenses/by/4.0/).

Butyrate, apart from inducing Tregs, also stimulates the activation of cytotoxic T cells (CTLs), leading to increased production and release of γ-interferon and granzyme, thus contributing to tumor eradication (Figure 1A) [18, 19]. Acetate is also active in this respect [20]. In addition to their effect on Tregs and CTLs, SCFAs impact natural killer (NK) cells and macrophages within the TME [21]. On the other hand, butyrate and propionate promote an anti-inflammatory environment, which may, under certain conditions, favor tumor progression [22]. These different and sometimes opposing effects of SCFAs underscore the complexity of immune modulation in the TME by the gut microbiota.

Two different bacterial metabolites of tryptophan (Trp) have profound immune-modulating effects relevant for tumor eradication. The first one is indole-3-aldehyde (I3A), generated by *Lactobacillus reuteri* (*L. reuteri*) (Figure 1B), which activates CTL, leading to increased γ-interferon production. In preclinical melanoma models, *L. reuteri* produced I3A improves the efficacy of ICI therapy [24]. Suggestive of the clinical relevance is the observation that the overall survival of melanoma patients on ICI therapy is better in patients with high serum levels of I3A [24].

Other gut bacterial Trp metabolites like indole-3-propionic acid (IPA), produced in tandem by *Lactobacillus johnsonii* (*L. johnsonii*) and *Clostridium sporogenes* (*C. sporogenes*) (Figure 1B), have demonstrated the ability to alter the epigenetic states of immune cells through mechanisms such as H3K27 acetylation [21, 25, 26]. In this context, the effect of IPA on so-called precursor exhausted T cells (Tpex) is relevant. Within TME, CD8^+^ CTLs are crucial mediators of antitumor immunity. However, after prolonged and persistent T cell receptor stimulation, they may differentiate into a dysfunctional state, termed exhausted T cells (Tex). However, Tpex cells can be rejuvenated to effector CTLs [27]. IPA can activate the transcription factor T cell factor 1 (TCF-1) by H3K27 acetylation at the super enhancer region of the *Tcf7* gene. TCF-1 induces Tpex activation and thus potentially enhances the effectiveness of ICI (Figure 1B). In an animal model, IPA is unable to enhance the efficacy of ICI therapy in *Tcf7*^-/-^ mice [26].

Gut bacteria have other direct or indirect effects (via metabolites) on the tumor cells and/or the TME, rendering tumors more susceptible to ICI therapy. Polyphenol-derived microbial metabolites, including hydroxyphenyl-γ-valerolactones (PVLs), can suppress the Wnt/β-catenin signaling pathway, a pathway commonly dysregulated in colorectal cancer (CRC) [22]. By altering cancer-associated fibroblasts (CAFs) and reducing tumor proliferation, polyphenol metabolites show promise as therapeutic agents that modulate the metabolic profile of the TME [28]. Gut bacteria can alter the metabolism of chemotherapeutic drugs such as irinotecan, affecting their efficacy and toxicity [29–31]. Organoid studies have revealed that microbiome manipulation induces phenotypic changes in tumor cells. These include altered expression of drug efflux pumps and anti-apoptotic pathways, which may lead to chemoresistance or chemosensitivity [32, 33]. Such findings emphasize the need for microbiome-targeted strategies to improve treatment outcomes. While *L. johnsonii* and similar microbes enhance immune therapies, identifying additional beneficial strains remains crucial [26] along with a better understanding of the mechanisms through which microbial metabolites enhance CTL activation, so that these attributes can be harnessed to improve immunotherapy responses across various cancer types.

Detailed investigations into specific microbial interactions with immune checkpoint molecules such as PD-1/PD-L1 could uncover novel ways to augment ICI therapies. Routy et al. [7] found that the effectiveness of ICI therapy, such as those targeting the PD-1/PD-L1 pathway, is influenced by the gut microbiome composition. For instance, the abundance of *Clostridioides* was found to be higher in melanoma patients who responded to ICIs, aligning with similar observations in the gut microbiome of responders. Conversely, *Gardnerella vaginalis* was more abundant in NRs [34]. Furthermore, the presence of certain bacteria, such as *Saccharopolyspora*, *Pseudoxanthomonas*, and *Streptomyces*, in pancreatic adenocarcinoma tissues has been linked to enhanced recruitment and activation of CD8^+^ T cells, a crucial component of the adaptive immune response against tumors [25, 35, 36]. This further supports the idea that specific bacterial taxa within the TME might modulate the efficacy of ICIs.

#### Optimizing and personalizing FMT

Originally, it was hypothesized that screening of the gut microbiota of patients who have responded favorably to ICI therapy could lead to the identification of universal FMT donors. Developing personalized FMT protocols tailored to individual microbiome profiles could optimize inflammation reduction, immune activation, and drug efficacy [37]. FMT, utilizing feces from a healthy donor, has demonstrated high success rates in treating recurrent *Clostridioides difficile* infection, with cure rates reaching up to 90%. This success stems from *Clostridial* infections being more responsive to microbial restoration via FMT. However, the efficacy of FMT in other (chronic) diseases such as inflammatory bowel diseases (IBD; ulcerative colitis and Crohn’s disease), irritable bowel syndrome, and metabolic disease has been less consistent, with clinical remission rates ranging from 24% to 50%. This variability has been attributed to IBD’s complex nature as a microbiome-driven immunological disorder influenced by host genetics and environmental factors [38, 39].

Dietary interventions could be an additional venue to optimize the effects of FMT. Dietary interventions aimed at promoting the production of beneficial microbial metabolites, such as polyphenols that modulate Wnt/β-catenin signaling, hold significant potential for CRC and other cancers [40]. Long-term dietary changes or supplementation with fibers and polyphenols could reshape gut microbiota composition, supporting anti-cancer metabolite production. Clinical trials assessing these interventions in combination with standard therapies are a critical next step. The first studies on the potential improvement of FMT success rate by dietary intervention showed mixed results. In patients with metabolic syndrome, the Mediterranean diet, neither for the donor nor the recipient, had no effect on the outcome of the FMT [41]. On the other hand, in ulcerative colitis patients, an anti-inflammatory diet did improve the outcome of FMT intervention on induction and maintenance of remission of the disease [42]. In an animal model of colitis-associated cancer (CAC), a high-fat diet was associated with reduced butyrate and accelerated CAC progression [43]. A number of studies on the effects of dietary intervention, either as a single intervention or combined with FMT, on the outcome of ICI therapy for cancer are ongoing [44].

### Local effects of bacteria in the tumor microenvironment

**Figure 2:**
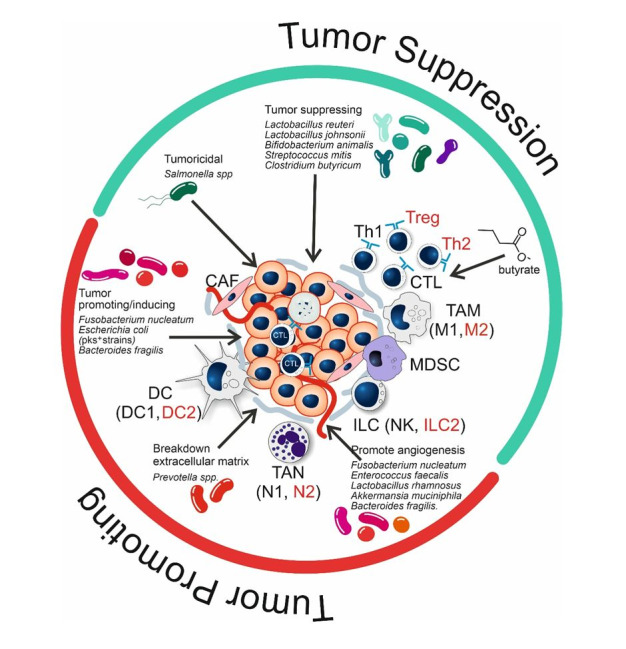
**Effect of bacteria and bacterial metabolites on the status (tumor suppression; green semicircle, or tumor promoting; red semicircle) of the tumor microenvironment (TME).** Cell types or differentiation states indicated in red have tumor promoting effects. Microbiota depicted in green have tumor suppressing effects; microbiota in red are tumor-promoting or -inducing (see text for further explanation). CAF: cancer-associated fibroblast; CTL: cytotoxic T cell; DC: dendritic cell; ILC: innate lymphoid cell; MDSC: myeloid-derived suppressor cells; NK: natural killer; TAM: tumor-associated macrophage; TAN: tumor-associated neutrophil; Treg: regulatory T cell.

As already indicated above, the TME consists of both malignant and non-malignant cells (including immune cells), the extracellular matrix (ECM), and soluble factors surrounding a tumor (Figure 2). Altogether, the TME influences the growth (either positive or negative) of the tumor and the outcome of treatment. Although these environments have classically been viewed as sterile sites, recent findings have uncovered the presence of certain bacteria residing within them (Figure 2). These bacteria can be distinguished from the microbiota on mucosal surfaces and have demonstrated important roles in the modulation of immune responses, behavior of tumors, and efficacy of therapies, including ICI.

Many studies have already established that bacteria can be found in different types of tumors, including those in the breast, lung, colon, and pancreas. With the use of 16S ribosomal RNA sequencing and microscopy, Nejman and co-workers [34] were able to profile over 1,500 tumors and found that most of them harbored bacteria, with the microbial compositions varying between the seven types of cancer that were investigated, and the microbial diversity being generally limited except for in breast cancer. Interestingly, bacteria were also found to be present intracellularly within both cancer and immune cells.

Recent research has also highlighted differences in bacterial composition and metabolism in tumor tissues compared to healthy tissues. In CRC, certain bacterial families, such as Lachnospiraceae, are found less in adenomatous polyps, while metabolites like acetoacetate and beta-hydroxybutyric acid accumulate. In cancerous tissues, an increase in bacteria such as *Fusobacterium nucleatum* subsp. *animalis* has been observed, along with the buildup of fumarate, a metabolite linked to altered cancer metabolism [45]. These findings suggest that specific bacterial populations and their metabolites may actively reshape the TME, potentially influencing tumor progression.

The location of a tumor is a significant factor that determines the likelihood of bacterial presence. Mucosal tumors, such as those in the lung or gastrointestinal tract, are directly exposed to external microbiota, increasing their likelihood of harboring bacteria [46]. In contrast, tumors found in otherwise sterile sites like the brain or pancreas may acquire bacteria through systemic circulation or transport by cells of the immune system. One mechanism by which bacteria translocate from the gut into the bloodstream is through disruptions of the intestinal barrier, making it more permeable, allowing bacteria to enter the systemic circulation and reach more distal sites. This can be caused by several factors, such as stress-induced ileopathy, chemotherapy, and high-salt diets [47–49]. Immune cells can also serve as vehicles for microbial transport. Dendritic cells (DCs) from the gut can capture bacteria and migrate to lymph nodes and distant organs, particularly when two ICIs are combined. Anti-PD-1 and anti-CTLA-4 therapy have been shown to activate the immune system and induce gut inflammation, disrupting normal barriers. This facilitates the translocation of specific gut bacteria, as DCs transport them into secondary lymphoid organs such as mesenteric lymph nodes and even tumors [50, 51]. Furthermore, the hypoxic conditions and leaky blood vessels typical of TMEs provide ideal conditions for the growth and colonization of anaerobic bacteria [52]. However, methodological challenges to accurately distinguish true intratumor bacteria from contamination, as well as the determination of the actual viability of detected bacteria, remain a challenge [34].

#### Systemic effects of bacteria on tumor cells and the TME

Bacteria can influence tumors not only through local interactions but also through systemic effects that are mediated by microbial metabolites. As discussed above, SCFAs and other bacterial metabolites that are produced in the gut can circulate throughout the body and modulate immune responses in distal tumors [53]. Additionally, the gut microbiome plays an important role in regulating systemic immune activation, which could impact the efficacy of ICIs in cancers outside of the gastrointestinal tract [54, 55].

Recent findings indicate that bacteria may also systemically influence metastatic potential by altering tumor cell metabolism. *Bacillus thermoamylovorans* was found to promote metastatic disease through the activation of enhanced glycolytic and nucleotide synthesis pathways, both of which have been associated with increased tumor aggressiveness [56]. In contrast, a related species, *B. subterraneus*, did not induce these changes. This highlights how functional bacterial genes, rather than bacterial presence alone, may drive disease progression. These findings suggest that systemic bacterial metabolites could selectively fuel metastatic dissemination and shape tumor evolution beyond the primary site.

#### Tumor suppression and tumor-promoting effects of microbiota in the TME

One of the key mechanisms through which bacteria within tumors exert local effects on the TME is immune modulation. Bacterial ligands can alter the behavior of tumor-associated macrophages (TAMs) to become more pro-tumorigenic, supporting the growth of tumors and suppressing immune responses [34, 57]. This is achieved through the activation of cell signaling pathways like NF-κB by the bacteria, which then activate the transcription of genes that drive inflammation and aid in tumor development [46]. However, not all intratumor bacteria promote cancer progression (Table 1). The oral administration of *S. mitis* isolated from gastric cancer tumors has been found to inhibit tumor growth, reducing tumor volume and extending survival in tumor-bearing mice [58]. This effect was mediated through the suppression of M2 macrophage polarization and infiltration, which reduced pro-tumor immune signaling.

**Table 1:** Tumor suppression and tumor promoting effects of microbiota.

**Bacterial species**	**Tumor suppressing**	**Tumor promoting**	**Reference**
*Akkermansia municiphilia*	-	PA	[59]
*Bacteroides fragilis*	-	PA, TP	[60]
*Bifidobacterium* spp.	TS	-	[61]
*Clostridium butyricum*	TS	-	[62]
*Enterococcus faecalis*	-	PA	[63]
*Escherichia coli* (pks+ strains)	-	TP	[55]
*Fusobacterium nucleatum*	-	PA, TP	[64]
*Lactobacillus johnsonii*	TS	-	[21]
*Lactobacillus reuteri*	TS	-	[24]
*Lactobacillus rhamnosus*	-	PA	[65]
*Prevotella* spp*.*	-	EM	[52]
*Salmonella typhi*	-	TP	[58]
*Salmonella* spp.	TC	-	[66]
*Streptococcus mitis*	TS	-	[58]

EM: degradation of extracellular matrix; PA: promotion of angiogenesis; TC: tumoricidal; TP: tumor promoting; TS: tumor suppressing.

Beyond the modulation of the immune system, certain bacteria actively modify important components of the TME to promote anti-tumor immune responses. *Salmonella* species have been demonstrated to exert varying effects on immune cells, tumor cells, and stromal components that promote tumor regression [66]. *Salmonella* can directly kill tumor cells by apoptosis, autophagy, pyroptosis, and ferroptosis. Through immunomodulation, *Salmonella* aids in recruiting and activating CTLs and NK cells and in reducing immunosuppressive Tregs and myeloid-derived suppressor cells. Additionally, the interaction of *Salmonella* with tumor stroma can lead to the remodeling of the ECM. This potentially impedes tumor invasion and metastasis. These findings suggest that besides playing a role in the modification of the immune landscape, *Salmonella* is also able to create a hostile environment that promotes tumor progression through the disruption of key structural and metabolic components of the TME.

Apart from *Salmonella,* other bacterial species also affect the structure and metabolism of the TME, facilitating tumor progression. *Prevotella* bacteria can change the composition of the ECM in such a way that it better facilitates tumor invasion and metastasis [52]. Other bacteria can also secrete angiogenic factors that alter the behavior of endothelial cells, which leads to the formation of new blood vessels that help sustain the growth of tumors [53, 57, 60]. These bacterial species include *A. muciniphila*, *B. fragilis*, *E. faecalis*, *F. nucleatum*, and *Lactobacillus. rhamnosus GG* (LGG) [59, 60, 63–65]. The angiogenic effects of *A. muciniphilia*, *B. fragilis*, and LGG are of interest because these species are either widely used probiotic bacteria (LGG) or otherwise associated with beneficial effects.

Certain bacteria have also been shown to directly interact with and stimulate oncogenic pathways. Certain strains of *Escherichia coli*, carrying the polyketide synthase (pks) operon, produce colibactin. This metabolite causes DNA damage and mutagenesis, thus contributing to the progression of CRC [55, 67]. In contrast, a direct reduction in tumor cell proliferation has been observed in mice treated with *S. mitis*, as indicated by a lowered expression of proliferation markers such as PCNA and Ki-67 [58]. Changes in the microbial composition within mouse tumors were observed after oral administration of this bacterium. This suggests that *S. mitis* can translocate from the GI tract to the site of the tumor, influence the structure of the TME, and impact tumor progression.

A recent study was able to identify distinct microbiomes and metabolomes in different stages of colorectal tissue transformation. This included bacterial families like Lachnospiraceae being depleted in adenomatous polyp environments and *F. nucleatum* subsp. *animalis* being more abundant in cancerous tissues [45]. These bacteria have been associated with the accumulation of beta-hydroxybutyric acid and fumarate within the TMEs, which may also contribute to the modification of the TME and colorectal tumor progression.

#### Implications for immune checkpoint inhibition therapy

The presence of tumor-associated bacteria significantly influences the efficacy of ICIs. In cases of esophageal squamous cell carcinoma (ESCC), enrichment of *Streptococcus* was associated with an increase in CD8^+^ T cell infiltration and a more favorable response to anti-PD-1 treatment [68]. Similarly, supplementation with *L. johnsonii* has been correlated with the upregulation of progenitor exhausted CD8^+^ T cells and enhanced effectiveness of anti-PD-1 immunotherapy in melanoma, breast cancer, and CRC [26]. In melanoma and lung cancer, a more diverse gut microbiome has been linked to better responses to ICI therapy [54]. However, some bacteria have also been found to reduce the efficacy of ICIs. *F. nucleatum*, a bacterial species commonly found in CRC and NSCLC, is associated with reduced survival and poor response to ICI therapy. This is potentially due to its role in promoting a more immunosuppressive TME through its inhibition of CTL activity [52]. Irrespective of ICI, Gammaproteobacteria and also *F. nucleatum* are able to breakdown gemcitabine, a common chemotherapy for pancreatic duct adenocarcinoma [29]. In those cases, antibiotic treatment prior to chemotherapy can improve the outcome and survival, but only from a median of 7 months to 14 months [69].

The growing insights into the roles of bacteria in the TME have paved the way for the development of more innovative combination therapies. Combining the targeting of the microbiota with ICIs could potentially optimize immune responses. Bacteria identified as immunosuppressive, such as *Fusobacterium*, could be eliminated with the use of antibiotics, while the abundance of bacteria identified as immunogenic, such as *Streptococcus* and *L. johnsonii*, could be increased by FMT from selected donors, by probiotics, or genetically engineered bacteria [34, 70]. It has been suggested that bacteria invading tumors at distal from the intestines are likely transported through systemic circulation via blood or lymph, or by immune cell transport. However, a therapeutic approach targeting these routes raises concerns about bacteria gaining access to other (healthy) tissues, which could lead to unintended side effects. A potential solution to this latter effect is engineering targeted delivery mechanisms that are capable of minimizing systemic side effects while optimizing the therapeutic effects of the tumor-associated bacteria.

Certain bacteria colonize hypoxic regions of the TME. *Clostridium novyi* (*C. novyi*)-NT targets hypoxic tumor cores, releasing toxins that selectively kill tumor cells. This forms the basis of bacteriolytic therapy, a promising experimental approach [29, 71]. Combining bacteriolytic therapies, such as those using *C. novyi-*NT, with chemotherapy, radiotherapy, or ICI therapy presents a promising avenue for treatment. Through genetic modification, other bacterial species could be created to preferentially target and colonize tumor tissues based on hypoxia.

Beyond influencing immune responses through microbiome composition, certain bacterial species may enhance the efficacy of ICIs by actively modifying the TME. *Salmonella*-based cancer therapies have been explored for their potential to synergize with ICIs [66]. By inducing pro-inflammatory signaling within the tumor, *Salmonella* enhances the infiltration and activation of cytotoxic immune cells, thereby increasing tumor sensitivity to checkpoint blockade. These effects suggest that *Salmonella* could serve as an adjuvant to ICI therapy, improving patient response rates while potentially overcoming resistance mechanisms. The ability of *Fusobacetrium nucelatum* to access human tumors has been demonstrated. This characteristic is also explored to be harnessed to use in cancer-targeting therapies [72]. One challenge is enhancing the specificity of the therapy to ensure that engineered bacteria target tumor tissue without affecting healthy organs.

The bacterial and metabolite composition of the CRC TME presents an opportunity to refine immunotherapy approaches. While a healthy gut microbiome and metabolome can help prevent malignancies, emerging research suggests that bacterial and metabolite signatures in precancerous adenomatous polyps may serve as valuable diagnostic markers for early CRC detection [45]. Additionally, leveraging TME composition to predict cancer progression and therapeutic response could help in the development of novel treatments and improve patient prognosis. Tumor-associated bacteria could be used as predictive biomarkers for therapy response. For example, a favorable response to chemoimmunotherapy could be predicted from the presence of *Streptococcus* in ESCC and *L. johnsonii* in melanoma, breast cancer, and CRC, while a resistance to ICIs could be predicted from high levels of *Fusobacterium* in colorectal and lung cancers [26, 52, 68]. The incorporation of microbiota profiling through tumor biopsy into the pre-treatment evaluation process for cancer patients could allow for personalized cancer treatment and improve the identification of patients more likely to benefit from ICIs. Depending on the tumor-associated bacterial species present, additional measures could also be taken to inhibit or enhance the patient’s microbiota.

## Conclusions

Despite advancements, significant challenges remain in studying the effects and impact of tumor-associated bacteria. The very existence of a TME microbiota could be questioned. Contamination during the processing of samples and the difficulty of distinguishing viable bacteria from fragments or dead cells complicate data interpretation [34]. Current experimental models are insufficient for studying low-biomass bacterial communities. For example, traditional mouse models lack the complexity of human microbiota and TME, necessitating the development of “triple-humanized” systems integrating human cancer, immune, and microbiota components [46].

Future studies should focus on further delineating the mechanisms by which bacteria influence tumor initiation, progression, and therapy resistance. Investigating the stability and evolution of tumor microbiota over time and under treatment pressures is a necessary first step in revealing the underlying mechanisms that ultimately determine interactions occurring in the TME [52]. The identification of metastasis-promoting bacterial genes could provide an opportunity to develop new therapeutic strategies that target bacterial metabolic pathways to disrupt pro-metastatic functions [56]. Therapeutic innovations, such as genetically engineered bacteria that could be targeted to the TME and release immune-stimulatory molecules or disrupt oncogenic pathways, have great potential for enhancing ICI efficacy [70].

It has been shown in controlled studies that modulation of gut microbiota composition and functionality by FMT can improve the outcome of ICI therapy. Large-scale implementation of this procedure is hampered by a number of factors, but mostly because the success rate for an individual patient cannot be predicted. The selection of so-called super donors for FMT selected from cancer patients successfully treated with ICI therapy has been troublesome because fecal material from these super-donors does not overcome ICI non-responsiveness in every patient. It should also be realized that if and when a universal super donor could be identified, large-scale application would still be limited. It is fair to conclude that at present, the exact mechanisms of success or failure of FMT cannot be predicted. Podlesny et al. [39] have shown that the level of engraftment of the FMT does not differ between responder and NR melanoma patients. Just as the devil is in the details, maybe the angels are also in the details. One of the YB328 bacterial strains could be the *Hominenteromicrobium* [73]. In a mouse model, this bacterium stimulates the migration of DC to the TME and promotes CTL function. Moreover, a favorable outcome of ICI therapy in patients carrying high numbers of YB328 has been observed [73]. These examples show that for future clinical practice, well-defined (consortia of) microbiota would be preferred in terms of safety, standardization, quality control, and ease of use. In a small phase 1 study of patients with metastatic renal cell carcinoma, progression-free survival was significantly longer in patients receiving ICI therapy with daily oral *C. butyricum* administration than without [62].

Pre-treatment microbiota profiling could also become a cornerstone of personalized cancer therapy, allowing for more precise patient stratification and treatment optimization [54]. Additionally, research into bacterial and metabolite signatures in precancerous adenomatous polyps could open new avenues for early CRC diagnosis. By identifying specific microbial and metabolic changes that precede malignancy, clinicians could implement screening tools for early detection and intervention, thereby improving patient outcomes and preventing cancer progression.

## Abbreviations

*A. muciniphila*: *Akkermansia muciniphila*
*C. novyi*: *Clostridium novyi*
CAC: colitis-associated cancer
CRC: colorectal cancer
CTL: cytotoxic T cell
DCs: dendritic cells
ECM: extracellular matrix
ESCC: esophageal squamous cell carcinoma
FMT: fecal microbiota transplantation
FOXP3: forkhead box P3
I3A: indole-3-aldehyde
IBD: inflammatory bowel diseases
ICI: immune checkpoint inhibition
IPA: indole-3-propionic acid
*L. johnsonii*: *Lactobacillus johnsonii*
*L. reuteri*: *Lactobacillus reuteri*
LGG: *Lactobacillus rhamnosus* GG
NK: natural killer
NR: non-responder
NSCLC: non-small cell lung carcinoma
PD-1: programmed death 1
PD-L1: PD-ligand 1
SCFAs: short-chain fatty acids
TCF-1: T cell factor 1
TME: tumor microenvironment
Tpex: precursor exhausted T cells
Treg: regulatory T cell
Trp: tryptophan

## Declarations

### Copyright

© The Author(s) 2025.
